# Electrophysiology of Hypothalamic Magnocellular Neurons *In vitro*: A Rhythmic Drive in Organotypic Cultures and Acute Slices

**DOI:** 10.3389/fnins.2016.00109

**Published:** 2016-03-31

**Authors:** Jean-Marc Israel, Stéphane H. Oliet, Philippe Ciofi

**Affiliations:** ^1^U1215, Neurocentre Magendie, Institut National de la Santé et de la Recherche MédicaleBordeaux, France; ^2^Université de BordeauxBordeaux, France

**Keywords:** burst firing, oxytocin, pulse generator, supraoptic nucleus, vasopressin

## Abstract

Hypothalamic neurohormones are released in a pulsatile manner. The mechanisms of this pulsatility remain poorly understood and several hypotheses are available, depending upon the neuroendocrine system considered. Among these systems, hypothalamo-neurohypophyseal magnocellular neurons have been early-considered models, as they typically display an electrical activity consisting of bursts of action potentials that is optimal for the release of boluses of the neurohormones oxytocin and vasopressin. The cellular mechanisms underlying this bursting behavior have been studied *in vitro*, using either acute slices of the adult hypothalamus, or organotypic cultures of neonatal hypothalamic tissue. We have recently proposed, from experiments in organotypic cultures, that specific central pattern generator networks, upstream of magnocellular neurons, determine their bursting activity. Here, we have tested whether a similar hypothesis can be derived from *in vitro* experiments in acute slices of the adult hypothalamus. To this aim we have screened our electrophysiological recordings of the magnocellular neurons, previously obtained from acute slices, with an analysis of autocorrelation of action potentials to detect a rhythmic drive as we recently did for organotypic cultures. This confirmed that the bursting behavior of magnocellular neurons is governed by central pattern generator networks whose rhythmic drive, and thus probably integrity, is however less satisfactorily preserved in the acute slices from adult brains.

## Introduction

The neurohypophyseal release of oxytocin (OT) and vasopressin (VP) by magnocellular neurons of the paraventricular and supraoptic (SON) nuclei is best achieved by action potentials (APs) emitted in bursts. This allows the secretion of a bolus of bioactive product (Poulain and Wakerley, 1982) and prevents secretory fatigue associated with continuous firing (Ingram et al., 1982). This feature made the magnocellular neuron system a remarkable model to study the mechanisms of stimulus-secretion coupling (Nordmann, 1983). A wealth of electrophysiological works (Brown et al., 2013) questioned the underlying mechanisms of the bursting activity displayed by the OT (high frequency bursts (HFBs) of APs) and the VP (phasic activity) neurons. These activities were alternatively attributed either to some intrinsic properties of these neurons, or to their synaptic afferent control.

Concerning the involvement of intrinsic properties of these neurons, it was suggested that the depolarizing after-potential (DAP) leads to sustained AP discharges in VP neurons (Andrew and Dudek, 1983; Armstrong et al., 1994; Ghamari-Langroudi and Bourque, 1998) while it sustains brief spike discharges in OT neurons (Armstrong and Stern, 1998). It was also suggested in OT neurons that the after-hyperpolarization (AHP) limits the firing of high frequency discharges (Teruyama and Armstrong, 2005), and that the hyper polarizing after-potential (HAP) generates a spike frequency adaptation (Israel and Poulain, 2000). Finally, we showed that some OT neurons display a low-threshold activated calcium current, presumably a T-current, responsible for the post-inhibitory rebounds triggering APs, and therefore up-regulating bursting activity in these neurons (Israel et al., 2008). A detailed account of the contributions of intrinsic properties to the bursting activity is available elsewhere (Armstrong et al., 2010).

Concerning the involvement of the synaptic afferent control in the generation of the bursting activity in magnocellular neurons, a detailed analysis at single-cell level was required, and *in vitro* models were preferred, and above all, the freshly prepared (acute) slices of the rat hypothalamus. Unfortunately, HFBs were only exceptionally observed in the OT neurons from acute slices (Israel and Poulain, 2015). This is not entirely surprising because these HFBs, triggering the release of OT for the milk-ejection reflex (Poulain and Wakerley, 1982; Brown et al., 2013), are provoked by the continuous suckling itself relayed to the OT neurons by sensory afferents that are inevitably severed when preparing acute hypothalamic slices.

In parallel to using the acute slice model (Israel and Poulain, 2000), we have therefore also explored another *in vitro* model, the organotypic culture of hypothalamic slices obtained from newborn rats (Gähwiler et al., 1978; Jourdain et al., 1996, 1998; Israel et al., 2010). In this model, which includes the SON and its adjacent areas, the OT and the VP neurons remain connected to their local intrahypothalamic regulatory networks allowing them to have an electrical activity similar to that seen *in vivo* (Israel and Poulain, 2015). Especially, OT neurons spontaneously display the coordinated HFBs of APs that are typically triggered by suckling in the adult lactating female rat, suggesting the existence and/or survival of a local glutamatergic burst-generator network in the organotypic culture (Jourdain et al., 1998). We very recently (Israel et al., 2014) provided evidence that this *in vitro* activity is physiological, due to a female-specific central pattern generator (CPG) whose rhythmic drive is visible when the raw electrophysiological recordings of the OT neurons are subjected to an autocorrelation analysis of the APs (AAA).

To further validate the hypothesis of a CPG drive of magnocellular neurons, as observed in cultured neonatal tissue, we have here reanalyzed using AAA our previous recordings obtained from the OT and the VP neurons in the model of the acute slice of adult hypothalamus. In parallel, we have also analyzed additional recordings from organotypic cultures. We confirm the hypothesis of a CPG drive in the acute slice model. However, in our hands, OT and VP neurons often appear disconnected from their respective CPGs in acute hypothalamic slices, whereas this connection generally appears preserved in the organotypic culture model. This paves the way to further *in vivo* explorations of cellular mechanisms of neuroendocrine pulsatility.

## Materials and methods

### Animals

Timed-pregnant, 10- to 12-week old, female Wistar rats (C.E.R.J., Le Genest Saint Isle, France) were received at G16 and housed in our animal facility (lights on from 0730–1930). Animals from acknowledged parturitions were used. The day of birth was designated lactational/postnatal day 1 (L1/P1), and litters were adjusted to 10 pups on L2/P2. For the acute slice experiments the dams were used at L5 or L21. For the organotypic culture experiments, female pups were used on P5, P7, P9, or P12. All efforts were made to reduce the number of animals used and any distress caused by the procedures, in strict compliance with the European Union recommendations (2010/63/EU) and as approved by the French Ministry of Agriculture and Fisheries (authorization number A33-063-090) and the local ethical committee of Bordeaux University.

### Acute slices

As detailed previously (Israel and Poulain, 2000), dams on L5 or L21 were anesthetized with 5% isoflurane and decapitated. The brain was quickly dissected out and immersed in ice-cold oxygenated (95% O_2_–5% CO_2_) perfusion medium for 1 min. The composition of the perfusion medium was as follows (in mM): 125 NaCl, 3 KCl, 1.24 MgSO_4_, 1.3 KH_2_PO_4_, 25 NaHCO_3_, 2 CaCl_2_, and 11 glucose. The brain was then blocked into a cube containing the bilateral hypothalamus, thalamus and medial amygdalae, the cube was fixed with cyanoacrylate glue on the top of the holder of a vibroslicer (MacIlwain; Campden Instruments, Lafayette, IN, USA) and three coronal slices (400 μm-thick) were cut and transferred onto a filter paper (optic lens neutral cleaner) in contact with a ramp-style interface recording chamber. The medium was perfused using a peristaltic pump (Gibson, Lexington, KY, USA) at a rate of 1 ml min^−1^ and at constant temperature (32 ± 0.5°C). The slices were kept oxygenated with humidified 95% O_2_–5% CO_2_. The electrophysiological session started following a 1.5 h-long equilibration period. The microelectrode was positioned into the SON with a micromanipulator (Microcontrôle) under visual control using a dissecting microscope. The electrode was lowered in 1 μm with a piezoelectric microdrive (Nanostepper).

### Organotypic cultures

Cultures were prepared using the roller tube method as described previously (Jourdain et al., 1996, 1998; Israel et al., 2010). Briefly, pups were anesthetized with 5% isoflurane and decapitated. Tissue blocks including the bilateral anterior hypothalamus and amygdalae were coronally sliced at 350 μm. The slices spanning the anterior hypothalamic area were selected, bisected along the third ventricle, the amygdalar tissue lateral to central/medial nuclei was removed, and each half was placed on individual glass coverslips coated with heparinized chicken plasma (Cocalico no. 30-030-5L, Reamstown, PA, USA) coagulated by drops of thrombin (Merck no. 112374). The coverslip was inserted into a plastic flat-bottomed tube (Nunc no. 055054, Roskilde, Denmark) containing 750 μl of a medium consisting of 50% Eagle's basal medium (Gibco no. 21540026, Fisher Bioblock, Illkirsh, France), 25% heat-inactivated horse serum (Gibco no. 26050088, Fisher Bioblock) and 25% Hanks balanced salt solution (HBSS; Gibco no. 24020091, Fisher Bioblock) enriched with glucose (7.5 mg ml^−1^) and 2 mM L-glutamine (Sigma no. G7513), pH 7.4 (290–295 mOsm). No antibiotics were used. The tubes were inserted into a roller drum at 37°C and rotated at ±15 turns.h^−1^. The medium was replaced twice per week. The cultures were allowed to flatten and stabilize for at least 4 weeks before electrophysiological recording became possible. For the recording session, the slice was transferred to a temperature-controlled chamber (36 ± 0.2°C) fixed to the stage of an inverted microscope (Diaphot; Nikon). The microelectrode was positioned using oleic micromanipulators (Narishige). The slices were perfused (0.7 ml.min^−1^) with Yamamoto's solution (in mM: 125 NaCl, 3 KCl, 1 MgSO_4_, 1.25 KH_2_PO_4_, 5 NaHCO_3_, 2 CaCl_2_, 5 glucose, 10 HEPES, pH 7.25, 293–295 mOsm. We performed our recordings of the VP neurons in cultures from P9 pups where they are more abundant (Jourdain et al., 1996, 1998; Israel et al., 2010). Hyperosmolality was produced by addition of mannitol.

### Electrophysiology

As detailed previously (Jourdain et al., 1996, 1998; Israel and Poulain, 2000; Israel et al., 2010), intracellular potentials (current clamp recording) from neurons were recorded through a single microelectrode (tip diameter: 0.1 μm) using an Axoclamp-2A (Molecular Devices, Union City, CA, USA), which also permitted injection of currents. Electrical signals were visualized on a digital oscilloscope (Tektronix TDS 2012B; Beaverton, OR, USA), recorded and stored on a hard disk using pClamp 9 software (Axon Instruments, Union City, CA, USA) and a Digidata 1300 interface (Molecular Devices, Sunnyvale, CA, USA). The AAA (Buchanan, 1999; Israel et al., 2014) was used to evaluate the periodicity as well as the regularity in neuronal activity. Episodes of OT and VP neuron activity of 3–5 min in duration were analyzed using custom scripts run within Spike2 software for Windows (CED, UK). Autocorrelograms were constructed from the spike trains of OT/VP cells converted to event times by setting a voltage threshold at half-amplitude of action potentials. For each event, the numbers of events occurring in consecutive 0.1-s bins were counted over a 20-s time period for OT cells and 60-s time period for VP cells. This procedure produced a periodic function with decaying oscillations. The periodicity of the rhythmic activity was obtained by measuring the lags from time 0 to the second peak on autocorrelograms. Measuring the peak-to-trough amplitude of the second peak on autocorrelograms assessed the Quality of the Rhythmic Activity (QRA) of OT/VP cells. Its value (denoted *a*) could potentially range from 0 to 1. A perfectly stable rhythm would have a QRA value of 1. Values are expressed as means ± SEM and analyzed using the Student's *t*-test with a significance level set at *P* < 0.05.

### Identification of recorded neurons

As detailed previously (Jourdain et al., 1996, 1998; Israel and Poulain, 2000; Israel et al., 2010), recording electrodes contained 0.5% biocytin (Sigma-Aldrich) injected at the end of the intracellular recording session using hyperpolarizing current pulses (±0.5 nA, 0.4 s, 2 Hz, 10–20 min). The tissues were fixed in a mixture of 4% paraformaldehyde and 0.15% picric acid in 0.1M phosphate buffer (PB) (acute slices, overnight at 4°C; organotypic cultures, 2 h at room temperature). Acute slices were also cryoprotected at 4°C in PB containing 20% sucrose, embedded in Tissue-Teck and sectioned (at 25 μm) in a cryostat (Microm, Francheville, France). Tissues then underwent triple-labeling immunocytochemistry including a first-step incubation with Texas Red-conjugated streptavidin (diluted 1:1000; Biosys, France), a mouse monoclonal antiserum to OT-associated neurophysin (diluted 1:2000; PS38 anti-OT-Np; ATTC No. ATCCRL1950, Rockville, MD, USA; kindly provided by Dr H. Gainer, NIH, Bethesda, MD, USA; see Ben-Barak et al., 1985) and either a polyclonal rabbit antiserum to vasopressin-associated neurophysin (VP-Np; diluted 1:2000; kindly provided by Dr A. Robinson, University of California at Los Angeles, CA, USA; see Roberts et al., 1991) or a polyclonal guinea pig antiserum to VP (1:800; Bachem No. T-5048; Bubendorf, Switzerland), followed by a second-step incubation with respective FITC-linked anti-rabbit IgGs and AMCA-linked anti-mouse or anti-guinea pig IgGs, all produced in goats or donkeys (Jackson Immunoreserach Europe, Newmarket, Suffolk, UK). Observation was with a Leica DMR epifluorescence microscope with appropriate filters (Leica Microsystems, Nanterre, France).

## Results

### Activity of OT neurons in acute hypothalamic slices

**Figure 1:**
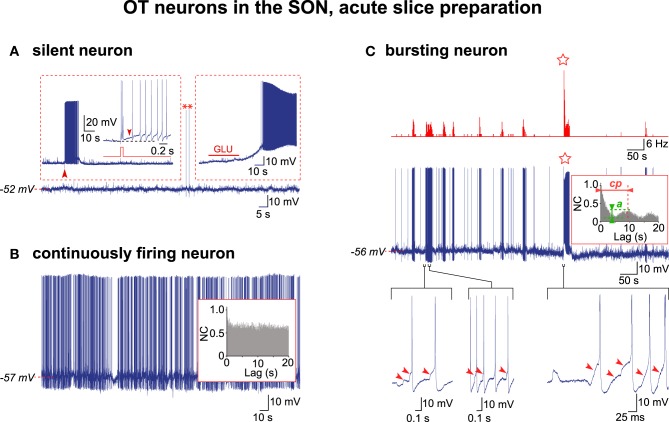
**Electrical activity in OT neurons in hypothalamic acute slices**. **(A)** A silent OT cell displaying rare action potentials (APs, asterisks) was subjected to two depolarizing challenges (insets). Left inset, a brief positive current pulse (50 ms, 0.1 nA; red trace; expanded from the large arrowhead) triggered a 10 s-long decaying burst of APs (the small arrowhead shows the DAP maintaining firing). Right inset, glutamate (GLU) in the perfusion medium (10^−5^ M, 30 s) induced a strong depolarization triggering a sustained firing. **(B)** A continuously active OT neuron with irregular firing with no rhythmic drive from the APs autocorrelation analysis (inset). **(C)** Exceptional example of a truly bursting OT neuron. Top, rate-meter showing the high-frequency burst (HFB) of action potentials (star). Middle, respective raw recording and associated autocorrelogram (inset) showing rhythmic drive. Bottom, expanded traces showing the importance of the EPSPs (arrowheads) in initiating the burst (left) and HFB (right) and triggering the APs within the burst (middle) (note that APs were truncated in the right trace).

We re-examined through AAA our previous recordings (Israel and Poulain, 2000) of OT neurons obtained in acute hypothalamic slices from lactating female rats taken on L5 or L21. Out of the 285 OT neurons recorded, each from a different SON section, only 67 cells displayed a stable resting membrane potential for more than 1 h and these were consequently selected for analysis (L5, *n* = 15; L21, *n* = 52). In acute slices from L21 rats, about 70% of the OT neurons (38/52 cells) remained silent in spite of a relatively depolarized resting potential (Figure 1A), the remaining neurons displaying continuous firing. In acute slices from L5 rats, most of the OT neurons (≈70%; 11/15 cells) recorded displayed continuous firing (Figure 1B). We selected ten of these continuously firing cells for AAA (L5, *n* = 3; L21, *n* = 7), which revealed no rhythmic drive in any of them (inset in Figure 1B). Of the 285 OT neurons recorded, an overwhelming majority (99.3%) never displayed HFBs of APs. Only two OT neurons from L5 slices displayed HFBs reminiscent of those described *in vivo* and in organotypic cultures (Figure 1C; Israel and Poulain, 2015). In these two bursting cells, trains of APs were initiated by a summation of EPSPs, the majority of the APs within a train were triggered by EPSPs, and a detailed analysis revealed that EPSPs initiated the HFBs (Figure 1C). The AAA was run on the recording of sufficient duration (320 s) of one of these two spontaneously bursting cells, which clearly revealed an activity supported by a CPG drive with a cycling period (cp = 9.7 s) and a quality of rhythmic activity (QRA; *a* = 0.23) similar to that seen (Israel et al., 2014) for the OT neurons in the organotypic cultures (insert in Figure 1C).

### Activity of OT neurons in organotypic cultures

**Figure 2:**
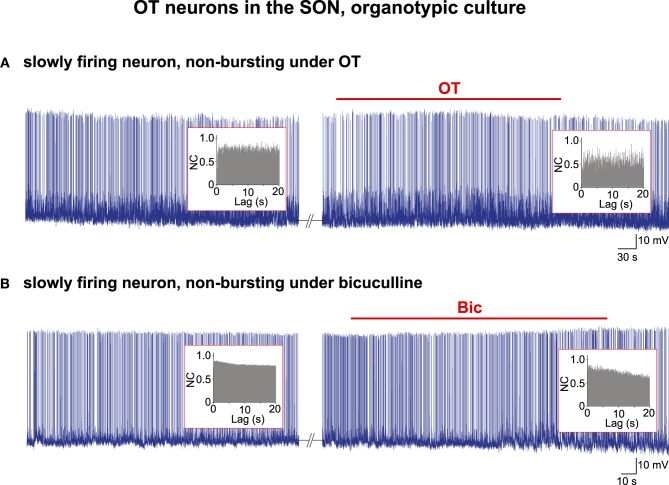
**Electrical activity in OT neurons in organotypic cultures**. Examples of two spontaneously firing cells that did not change their pattern of activity under bath application of **(A)** the OT peptide (10^−5^ M) or **(B)** the GABA_A_ receptor antagonist bicuculline (Bic; 10^−5^ M). No rhythmic drive was detected by the AAA (insets) either before or during the application of the burst-inducing agents.

Briefly, as reported recently (Israel et al., 2014), in about 90% of the organotypic cultures, all the OT neurons burst in a highly coordinated manner and their individual activity according to AAA appears to be driven by a CPG network (96/107 cultures from P5 animals examined harbored bursting OT neurons). However, in the remaining 10% of the cultures, the OT neurons do not display HFBs of APs, nor qualify for a rhythmic drive when their activity is subjected to the AAA. When these neurons are challenged with the OT peptide (*n* = 6; Figure 2A) or the GABA_A_ receptor antagonist bicuculline (*n* = 6; Figure 2B) to induce robust bursting (Jourdain et al., 1998; Israel et al., 2014), their activity remains unchanged and the AAA reveals no rhythmic drive either before or during the challenges (Figure 2).

### Activity of VP neurons in acute hypothalamic slices

**Figure 3:**
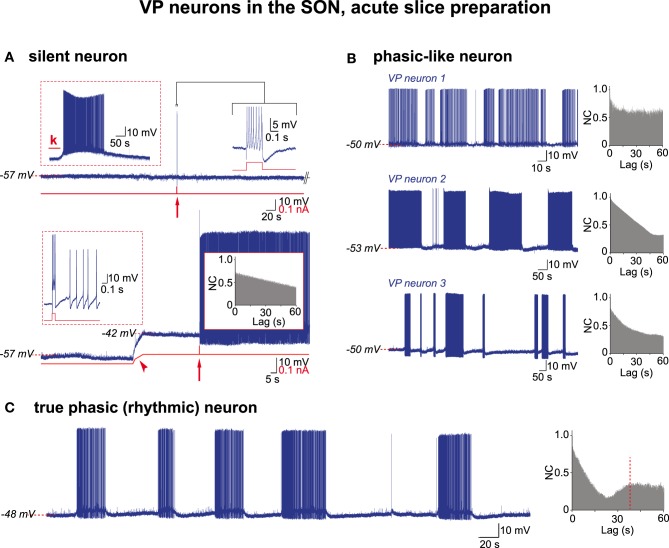
**Electrical activities in VP neurons in hypothalamic acute slices. (A)** A silent VP cell displaying action potentials only if subjected to depolarizing challenges. Top trace, left inset, sustained firing in response to kainate (k) in perfusion medium (10^−5^ M; 30 s); right inset, a train of APs (expanded trace; truncated) in response to a brief positive current pulse (0.2 s, 0.15 nA; red arrow). Bottom trace, the same neuron when depolarized by constant current injection (red trace, arrowhead) now displays a prolonged firing in response to a brief current pulse (0.1 s, 0.1 nA) (left inset, expanded from arrow). Note absence of rhythmic drive (right inset). **(B)** Three examples of phasic-like activities displayed by VP neurons. Neuron 1, bursts and silent periods of irregular duration. Neuron 2, bursts and silent periods of similar duration. Neuron 3, short and long bursts, irregular silent periods. Autocorrelograms show no rhythmic drive. **(C)** A truly phasic activity sustained by a rhythmic drive.

**Figure 4:**
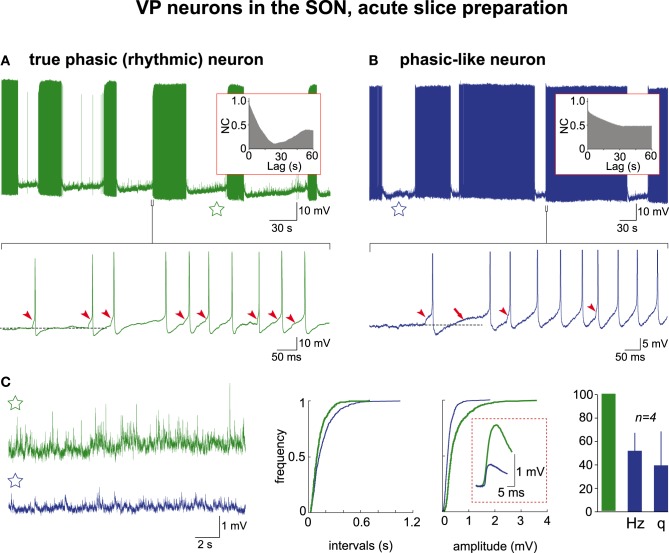
**Electrical activities in VP neurons in hypothalamic acute slices**. Mechanisms of burst generation in spontaneously active neurons **(A)** with and **(B)** without rhythmic drive (autocorrelograms in the insets). The raw recordings (upper traces) are expanded (lower traces) to reveal that the first AP in the burst is triggered by an EPSP (arrowheads) and is followed by a DAP (arrow) in **(B)** only (APs are truncated in **B**). The subsequent APs in the burst are essentially triggered by EPSPS in **(A)** and by both EPSPs and DAPs in **(B)**. Note in the samples of inter-burst activity (stars) magnified in **(C)** the heightened synaptic activity in **(A)** reflected by the cumulative frequencies of EPSPs' intervals and amplitudes (mean amplitudes in dashed frame). The histogram shows that phasic-like neurons display EPSPs at lower frequency (Hz) and smaller amplitude (q) (green column = 100% for both frequency and amplitude of events recorded in truly rhythmic neurons; *n* = 4 each).

In the following, by convention, the activity of VP neurons will be referred to as truly phasic when the AAA revealed a CPG drive, and phasic-like when the AAA did not. We re-examined through AAA our previous (Israel and Poulain, 2000) recordings of VP neuron activity in acute hypothalamic slices from nursing female rats taken on L5 and L21. These magnocellular neurons were identified *post-hoc* as VP neurons each recorded from a different SON section (L5, *n* = 21; L21, *n* = 57). Irrespective of lactation day, about half (37/78 cells; 47%) of the VP neurons had a low resting membrane potential close to −58 mV and remained silent, displaying only a few spontaneous APs (Figure 3A). These cells were healthy as they generated a brief burst of APs in response to a short electrical stimulation, a robust firing following bath application of glutamate, and a continuous activity when depolarized above spike threshold (Figure 3A). When these various patterns of activity were subjected to the AAA, no rhythmic drive was detected (*n* = 4 cells for glutamate pulse; *n* = 10 cells for depolarization-induced firing). The remaining population (41/78 cells) of VP neurons had a more depolarized resting membrane potential (≈−50 mV) and spontaneously displayed phasic-like activity (Figure 3B). There was a great cell-to-cell heterogeneity in the firing patterns that were made of irregular long bursts (10–200 s in length) interspersed with silent periods of varying duration (10–300 s in length) (Figure 3B), and two sub-populations clearly emerged with respect to their mean burst-duration (20–50 s and 75–150 s). When subjected to the AAA, only the short-burst (20–50 s) population qualified for a CPG drive (cp = 41.3 ± 16.1 s; a = 0.36 ± 11) (short-burst *n* = 8; long-burst, *n* = 17) (Figures 3C, 4) and this population also exhibited a more robust excitatory synaptic activity (Figure 4C). It is noteworthy that in these neurons APs were essentially triggered by EPSPs (Figure 4A, lower trace).

### Activity of the VP neurons in organotypic cultures

**Figure 5:**
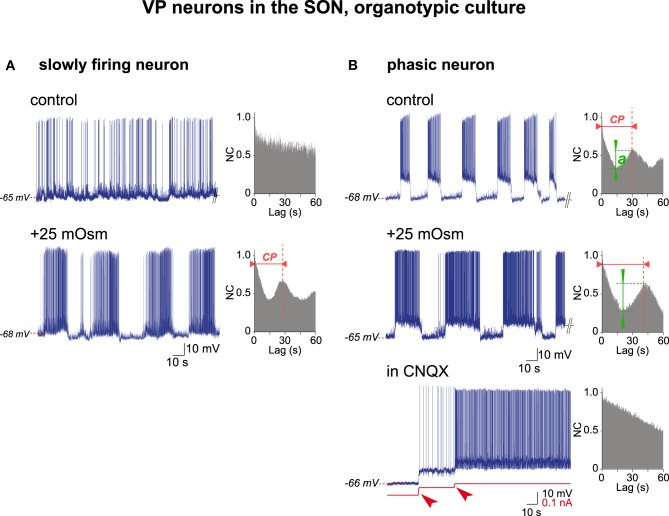
**Electrical activities in VP neurons in hypothalamic organotypic cultures**. **(A)** Transition from irregular (top, control medium) to phasic (bottom, +25 mOsm medium) activity in a VP neuron by a hyperosmotic stimulus. Note rhythmic drive [with a cycling period (*c.p*.) of 28 s] in phasic firing-mode only. **(B)** Increase in rhythm quality in a spontaneously phasic VP neuron (top, control medium) by a hyperosmotic stimulus (middle, +25 mOsm medium) and the dependence of phasic firing on glutamatergic inputs (bottom). Note that hyperosmolality increases the cycling period (*c.p*.) and the quality of the rhythm (*a*), while CNQX (10^−6^ M) abolishes spiking and rhythmic drive (depolarization by two successive current injections (arrowheads) induces spontaneous firing not supported by a rhythmic drive).

The electrical activity of VP neurons in organotypic cultures was re-examined through AAA. To this end we used the VP neurons identified in our previous studies carried out in organotypic cultures (Israel et al., 2010, 2014), representing a total of 147 VP neurons each recorded from a different culture. Among those, 67% (98/147 cells) spontaneously displayed a slow and irregular firing pattern (varying from 0.5 to 10 Hz) and the AAA (*n* = 6) revealed that this activity had no underlying rhythmic drive (Figure 5A). Increasing the osmolality from 295 to 320 mOsm (hyper-osmotic stimulus; +25 mOsm) in all cases turned this slow firing pattern into a typical phasic activity that the AAA clearly identified as a sequence of rhythmic oscillations with a cycling period of about 30 s (mean ± SEM; 33.7 ± 3.9 s; *n* = 6; Figure 5A). The remaining VP neurons (33%; 49/147 cells), in our iso-osmotic conditions (295 mOsm), spontaneously displayed phasic activity, made of more or less regular bursts interspersed by silent periods, that the AAA again identified in all cases as a rhythmic firing pattern with a cycling period varying from 12 to 58 s (31.7 ± 12.1 s; mean ± SD; *n* = 15 neurons; Figure 5B). When spontaneous (at 295 mOsm) and induced (by 320 mOsm) phasic activities were compared, the quality of the rhythmic activity (or QRA) was higher in hyper-osmotic medium [spontaneous (*n* = 5) 0.23 ± 0.05 vs. induced (*n* = 5) 0.39 ± 0.08; mean ± SD; *P* < 0.001; Student's *t*-test]. Finally, in all cases the phasic activity, either spontaneous or induced, was blocked by the AMPA-kainate receptors antagonist CNQX, supporting the notion that it is determined by glutamatergic afferent synaptic inputs rather than by an intrinsic neuronal property (Figure 5B). All these results are summarized in Table 1.

**Table 1:**
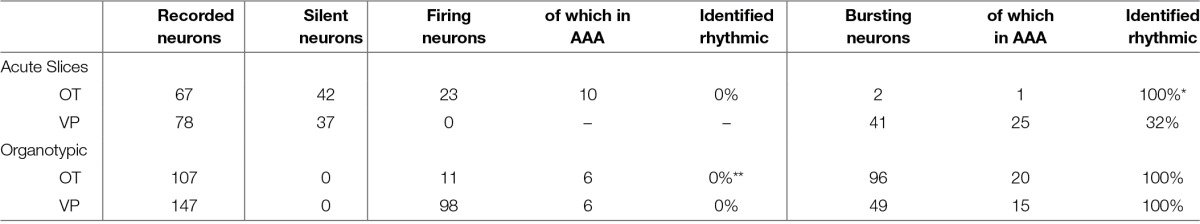
**Electrophysiological activity of hypothalamic magnocellular neurons ***in vitro*****.

^*^Non representative, exceptional instance of bursting in 2/285 neurons studied (of which 67 recorded); ^**^From the 10% of the cultures with no bursting neurons. See text.

## Discussion

Our own previous (Jourdain et al., 1996, 1998; Israel and Poulain, 2000; Israel et al., 2010, 2014) data have now been reconsidered in the light of our recent hypothesis on the existence of specific CPGs driving neuroendocrine magnocellular neurons (Israel et al., 2014). These data suggest that if the HFBs of APs that are typical of these secretory cells are well visible in the organotypic culture model, such a CPG-driven rhythmic activity is also present, even if not as commonly observed, in acute slices. There is a straightforward, albeit not exclusive, explanation for the lack of a strict parallelism between the outcomes of the two *in vitro* models, especially concerning OT neurons. Presumably, an anatomical link between the CPGs and the magnocellular neurons is severed during the preparation of acute slices, whereas this link is preserved, at least in part, during the harvest of the tissue from the newborns (Israel et al., 2014). Conceivably, because neonatal and adult brains were cut at the same standard thickness of about 350–400 μm, more neurons, and therefore more networks, are harvested in the pups compared to the adults, and as a consequence, the networks and their emerging properties that govern the secretory activity of magnocellular neurons will be more often observed from neonatal preparations.

In the acute slice model, the bursting pattern of the OT neurons that is necessary for their motor output (contraction of the myometrium and the mammary myoepithelial cells) was seen in only 2 out of 285 cells, that roughly corresponds to 1% of the (bilateral) slices collected. Considering the anatomical distance in adult brains between the SON in the one hand, and the posterior periventricular area that seems determinant for burst generation (Israel et al., 2014) in the other hand, it is likely that 99% of the slices have a significantly deafferented SON. In line with this possibility, the synaptic activity in acute slices is dramatically reduced to miniature events (Israel and Poulain, 2015), suggesting that in our hands most of the afferent axons including those of the CPG network are severed during the preparation of these slices.

Likewise in the acute slice model a number of the VP neurons appear to have no link to a CPG drive, because their activity do not qualify for periodicity as revealed by the AAA. Yet, a significant proportion of them spontaneously displayed as here defined a phasic-like activity (long-burst neurons). Most probably, this phasic-like activity is essentially due to an intrinsic property of the VP neurons, the depolarizing afterpotential (DAP) (Andrew and Dudek, 1983; Sabatier et al., 2004), as it could be observed in the acute slices even when synaptic transmission is blocked (Hatton, 1982). In this phasic-like firing mode, successive DAPs establish a plateau membrane potential bringing the cell to firing threshold; this is observed when the cells are quite depolarized (Armstrong et al., 1994) with respect to their normal resting value (Figure 3A, lower trace), and consequently fire APs spontaneously. In organotypic cultures, 60% of VP cells displaying a spontaneous phasic activity did not show DAPs (see Figure 7B in Israel et al., 2010) and pharmacological blockade of the latter did not significantly alter the phasic pattern (Israel et al., 2010). Again, DAPs were ineffective at generating a sustained firing in cells at their normal resting potential (near -50 mV; see Figure 7E in Israel et al., 2010). Thus, this non-periodic, phasic-like activity is probably non-physiological and can also be evoked in OT neurons (Israel and Poulain, 2015). Accordingly, we previously showed (Israel et al., 2010) that the various parameters of this phasic-like activity (burst duration, mean intra-burst firing frequency, mean burst duration and mean silent duration) differ from those displayed by the VP neurons *in vivo* or in organotypic cultures. Indeed, in most acute slices the synaptic activity recorded from magnocellular neurons was low and the AAA did not detect any underlying rhythm. Conversely in the remaining slices, VP neurons displaying short bursts of APs and a phasic firing mode clearly supported by a CPG also expressed a more robust synaptic activity. These short-burst VP neurons are the only type to be observed both *in vitro* and *in vivo* (see Figure 4 in Israel et al., 2010) and their activity is thus likely to correspond to a physiological behavior. In these cases, local CPG networks present in the surroundings of the SON (Brown et al., 2013) most probably remained intact at tissue harvest. This fits with data from *in vivo* studies in which glutamate receptors antagonists blocked the phasic activity in the VP neurons (Nissen et al., 1994, 1995; Brown et al., 2004), clearly suggesting the critical role of the afferents to these cells.

By studying a large series of organotypic cultures, we noticed that about 10% of these cultures harbored healthy, spontaneously firing, but non-bursting OT neurons (Israel et al., 2014). These OT neurons did not change their activity when challenged with the OT peptide itself or with the GABA_A_ receptor antagonist bicuculline, two procedures known to elicit strong bursting activity (Jourdain et al., 1998; Israel et al., 2014), and when analyzed through AAA, their activity either before or during the challenge, accordingly showed no rhythmic drive. This suggests that the OT-CPG network is absent in about 10% of the cultures, compared to 99% for acute slices obtained in our hands from adult brains.

**Figure 6:**
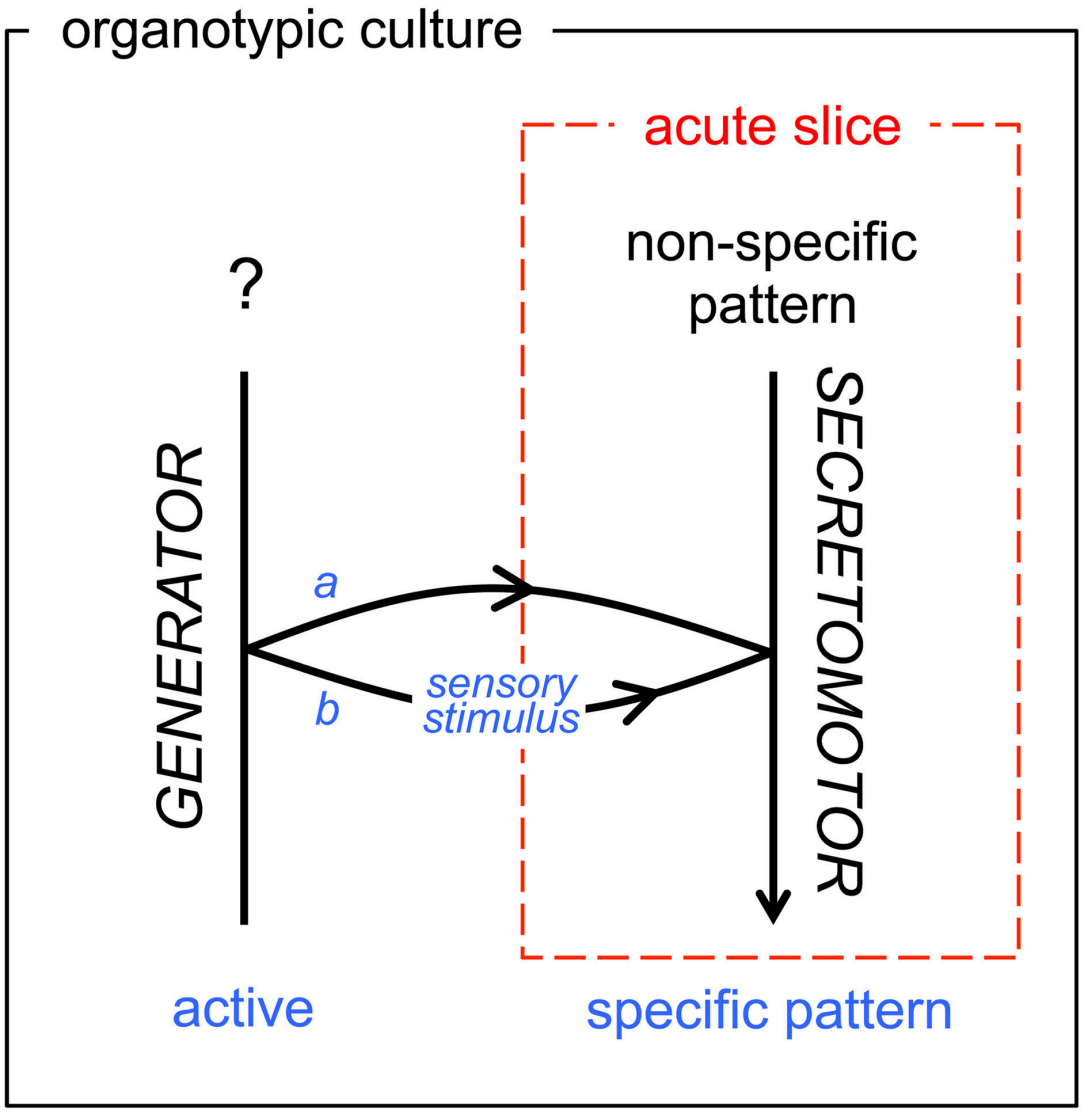
**Schematic diagram of functional state transition of the secretomotor OT and VP neurons ***in vitro*****. As visible in our acute slice model (dashed red frame) and in our organotypic culture model (black frame), the transition from random firing to the typical neurosecretory bursting/phasic pattern is either inherent (*a*, developmentally active: OT neurons) or gated (*b*, elicited by sensory stimuli: VP neurons). The acute slice model in most cases (99% for OT neurons, 70% for VP neurons) does not include the generator network. See Conclusion.

The organotypic culture model, here, allowed us to get a deeper insight into the mechanism generating the phasic activity in the short-burst VP neurons. The phasic activity was spontaneously displayed or could be evoked by a hyperosmotic challenge. In both cases, the rhythmic pattern of activity could be blocked with a glutamate receptors antagonist, and appeared driven by a rhythmic network as revealed by AAA. In contrast, the sporadic activity of the short-burst VP neurons before hyperosmotic challenge showed no underlying rhythmic drive when subjected to AAA. Altogether, these observations suggest that the osmotic stimulus gates the CPG activity to the effector neuroendocrine cells (Figure 6), although a precise mechanism remains to be fully explored.

## Conclusion

### Contributions of the experimental models

Both experimental models used here have inherent limitations that must be borne in mind when interpreting our observations and delineating their impact. The acute slice model is a strict reflection of the adult organization of the networks, but the recorded cells are partially deafferented. The organotypic model offers a more stabilized preparation, but a number of regulatory networks are certainly not in place at the time of tissue harvest. In this case, the spontaneous bursting recorded in OT neurons may reflect the autonomous activity of a dedicated neural network (referred to as OT-Central Pattern Generator, OT-CPG) producing fictive neurosecretory activity, in a way similar to the fictive motor patterns produced by CPGs located in the brainstem or spinal cord (Grillner, 2003). Therefore, the acute slices and the cultures cannot be strictly compared (Figure 6), especially when the role of the integrative properties of the magnocellular neurons are to be considered.

Our data, gathered *in vitro* and *ex vivo*, suggest that afferent rhythmic CPG networks govern the high frequency activity in OT and VP neurons. Our data also suggest that although the VP-CPG and OT-CPG networks are quite different, their functional output, the bursts of APs triggering the release of the neurohormones, is essentially the same. These considerations concerning the sole magnocellular neurons illustrate the progressively appreciated diversity of the mechanisms underlying the activity of neuroendocrine systems, as discussed below.

### Contributions to the understanding of the activity of neuroendocrine systems

Most of what is known of the *in vivo* electrophysiological activity of neuroendocrine cells is derived from the study of the magnocellular hypothalamo-neurohypophysial neurons (Brown et al., 2013). These neurons are fine models for the study of the stimulus-secretion coupling, and their episodic firing, at a high frequency necessary for neurosecretion, appears to be a universal mechanism of peptidergic neurotransmission (Hökfelt et al., 2003). It is therefore expected that to trigger a secretory pulse of anterior pituitary hormone, the parvicellular adenohypophysiotrophic neurons have a similar activity. This, however, is still unknown, due to the overwhelming difficulty of *in vivo* “blind” recording of parvicellular neurons: they have a smaller size, are more dispersed, their various sub-populations often intermingle, and the occurrence of a pulse of anterior pituitary hormone in basal physiological conditions is less immediately accessible than, for example, reflex-milk ejection.

Yet, it was early observed *in vivo* that every pulse of anterior pituitary luteinizing hormone (LH) strictly correlates with volleys of high frequency multiunit electrical activity (MUA volleys) in the mediobasal hypothalamus (Knobil, 1999). These MUA volleys are thought to reflect the activity of the afferent network responsible for the pulsatile release of gonadotropin-releasing hormone (GnRH), the so-called GnRH pulse-generator (Ezzat et al., 2015). The notion discussed here that CPGs drive secretory OT and VP neurons, thus recalls the presumptive organization of the network responsible for the pulsatile secretion of LH.

In the case of pituitary prolactin secretion, a different mechanism seems to be at work, the hormone being under tonic inhibitory control by dopaminergic tubero-infundibular (TIDA) neurons; a constant release of inhibitory dopamine keeps prolactin secretion low, with transient interruptions of this neuroendocrine tonus allowing pulsatile secretion (Grattan, 2015). Recent *ex vivo* studies in the rat have analyzed the electrical activity of TIDA neurons in acute slices and subjected the recordings to an AAA (Lyons et al., 2010). It appeared that TIDA neurons in control conditions display a synchronized robust, high-quality rhythmic activity dependent upon gap-junction coupling. In this case, an afferent CPG drive is not necessary to generate a rhythmic behavior, which is an emerging property of the neuroendocrine TIDA cell network. However, it was also shown that the sole direct post-synaptic action of thyroliberin could induce TIDA neurons to abruptly switch from rhythmic to tonic firing mode. This suggests that modulatory afferent networks control abrupt transitions in functional state, recalling what we saw in organotypic cultures for VP neurons switching from irregular to true phasic activity under hyperosmotic challenge (Figures 5, 6).

The pulsatile ultradian secretion of corticotrophin (ACTH) does not appear to depend on a rhythmic hypothalamic neuroendocrine drive. A strong corpus of physiological studies together with mathematical modeling indeed suggests that a sub-hypothalamic feedforward/feedback (ACTH/corticosterone) endocrine oscillator is sufficient to explain pulsatility (Russell et al., 2015). In this case, the global hypothalamic drive through the neurohormone corticoliberin (CRH) would rather be tonic/permissive than phasic/causal, and the associated electrical activity of CRH neurons remains to be established.

Altogether, these data suggest that the hypothesis of an exclusive CPG drive of follower neuroendocrine cells, such as that suggested by our studies, may not be generalized to every neuroendocrine axes or at least not to every circumstances of their activation. This would fit with the diversity in the modalities of the hypothalamic neuroendocrine control of the anterior pituitary. Nevertheless, a clearer picture concerning magnocellular and parvicellular neurons will be provided by AAA of their *in vivo* activity in adult animals with a monitored pituitary output. Of particular interest would be situations expected to require functional state transitions (Figure 6) to produce large secretory responses such as osmotic/hemorrhage challenge, stress-induced ACTH release, suckling-induced prolactin/OT release, or pulse/surge of LH. This would allow a better understanding of the contribution of endogenous and network properties to the adaptive responses of these cells. An excellent thorough discussion of this topic is available elsewhere (Lyons and Broberger, 2014).

### Contributions to the understanding of the organization of the hypothalamus

**Figure 7:**
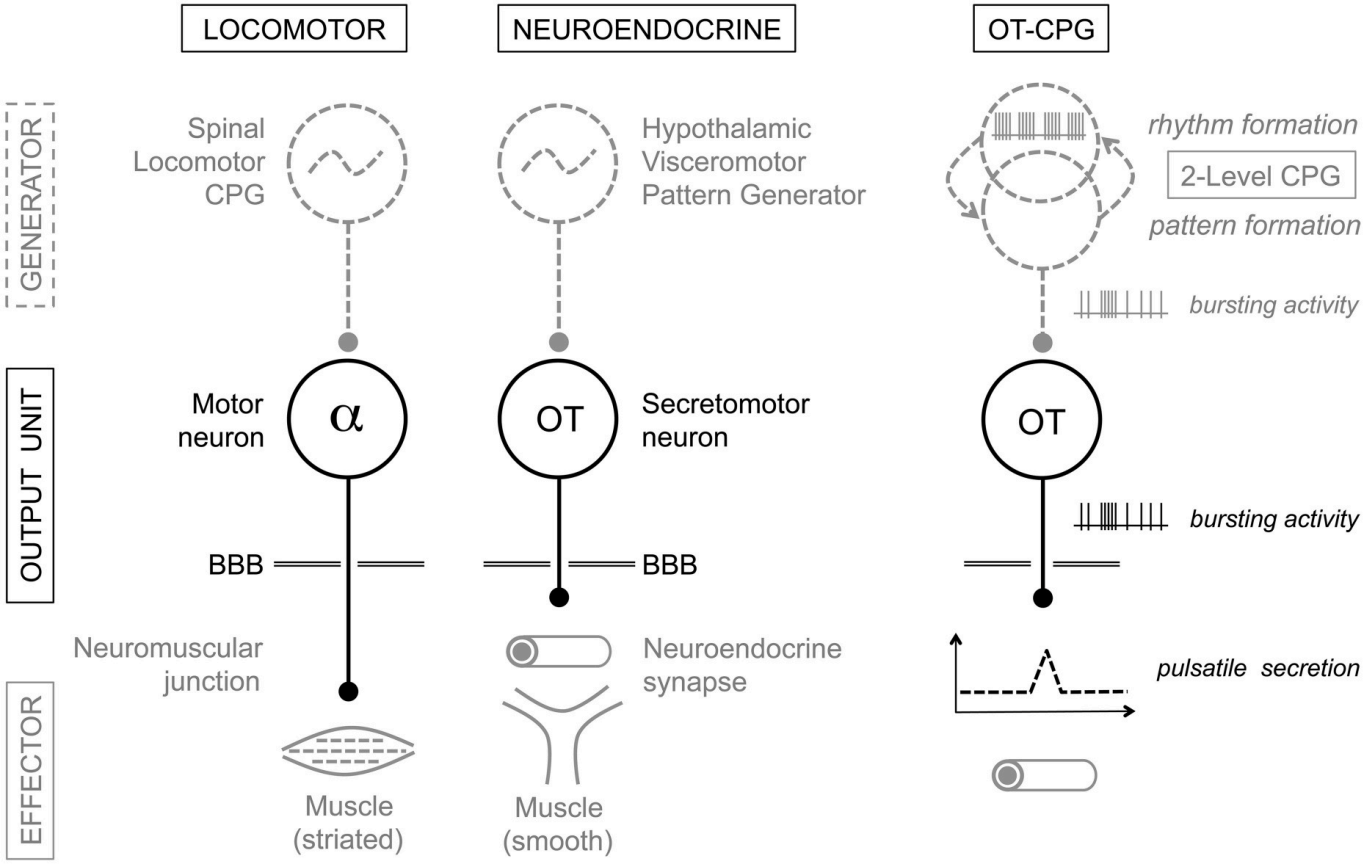
**Analogy between the locomotor and neuroendocrine circuits**. Motor neurons and neuroendocrine cells are output units driven by central pattern generator (CPG) networks. CPGs generate the specific electrical firing required for secretion of neurotransmitters/neurohormones and the desired action of the effector structure: skeletal muscle for alpha-motor neurons, uterus and mammary myoepithelial cells in the case of magnocellular oxytocin (OT) neurons. The presumptive “OT-CPG” necessary for the milk-ejection reflex may be a two-level CPG comprising a rhythmogenic component in interaction with a pattern-forming component, the output of which being bursting activity. The secretomotor unit is mainly a follower of the CPG output triggering neurosecretion. BBB, blood-brain-barrier. For details, see the Conclusion.

Neuroendocrine cells are the final output neurons in their networks and have their targets outside the blood-brain-barrier. This arrangement early prompted an analogy with the output units of the locomotor circuits, i.e., the alpha motor neurons in the spinal cord (Harris, 1955; Markakis, 2002; Watts, 2015; Figure 7). More recently (Thompson and Swanson, 2003), an extensive analysis of intra-hypothalamic connectivity has proposed the existence of a meta-network responsible for the coordination of neuroendocrine and behavioral systems. This proposed “hypothalamic visceromotor pattern generator” (HVPG) network includes an extended CPG hosted by the rostral half of the periventricular continuum (PeV). As remarked by the authors, this disposition of the HVPG along a liquor compartment and near its motor targets indeed recalls the similar location of the spinal locomotor CPG along the central canal (Grillner, 2003; McCrea and Rybak, 2008), which reflects current efforts to comprehend the basic organization of the forebrain (Croizier et al., 2015).

The concept of a HVPG thus makes the testable prediction of the existence of rhythmic networks in the PeV, which we have indirectly met. Indeed, by removing the PeV from the tissue slices harvested for organotypic culture, we rendered OT neurons non-bursting and their afferent activity non-rhythmic in AAA (Israel et al., 2014). This indicates that at least part of the OT-CPG resides in the PeV, but provides little information on its architecture. The responses of OT neurons in culture to inhibition-blockade by bicuculline and excitation-blockade by CNQX (Israel et al., 2014) further suggest that the organization of the OT-CPG resembles the “two-level organization” proposed for the spinal locomotor CPG (Perret and Cabelguen, 1980; McCrea and Rybak, 2008) and the brainstem respiratory CPG (Feldman et al., 1990). Indeed, bicuculline transformed bursting activity into robust oscillations, revealing a glutamatergic rhythm generator entirely blocked by CNQX, as well as revealing a GABAergic pattern formation circuitry responsible for OT activation (Figure 7). Concerning the VP-CPGs in culture, neither PeV removal nor bicuculline impacted rhythmic activity in VP neurons (Israel et al., 2014). This indicates that the VP-CPGs are separate from the OT-CPGs, can work independently from the periventricular HVPG, and likely are “satellite systems” of the magnocellular nuclei (Bourque, 2008). Our studies have however provided little insight into their architecture, precise location, and functional interplay with the cellular properties of VP neurons.

### Future directions

Our present studies in acute slices and organotypic cultures constitute an initial effort to decrypt some structure-function relationships in the magnocellular neuroendocrine circuits, complementing current efforts to decipher the electrical behavior of hypothalamic neuroendocrine cells (Osterstock et al., 2010; Murphy et al., 2012; MacGregor and Leng, 2013; Wamsteeker Cusulin et al., 2013; Ohkubo et al., 2014; Briffaud et al., 2015; Iremonger and Herbison, 2015; Royo et al., 2015). Although, this ideally at some point requires analysis and manipulations of the neuroendocrine networks *in vivo* (Campos and Herbison, 2014), a wealth of *ex vivo* studies should also continue to provide irreplaceable information.

To further progress in that direction concerning neuroendocrine magnocellular neurons, it seems important to confirm and extend our *ex vivo* observations in acute tissue slices from adult animals, at least for VP neurons and their response to an osmotic challenge. More difficult to predict is how to preserve in acute slices of lactating female rats the putative PeV rhythmic afferent drive of OT neurons. This may require the investigation of various orientation planes of the slices, e.g., as done for GnRH neurons (Constantin et al., 2012), but without guarantee. Indeed, our very low success rate with this acute model may also be indicative of the difficulty to maintain the bursting of OT neurons in an abruptly deafferented adult network that was functionally activated by suckling, as opposed to an incompletely afferented, immature and autonomously active OT-CPG core network after several weeks of recovery *in vitro*. However, there might be permanent ongoing activity in the PeV continuum that could be recovered from acute slices sagittaly cut along the third ventricle, as predicted (Thompson and Swanson, 2003). It may be worth searching for this in rat hypothalamic slices loaded with a calcium indicator, or at best in murine transgenic models. Ideally, one may want to perform in parallel, or combined, electrophysiological recording and calcium imaging in specific models. For example, mouse lines could be engineered and crossed for having, neurons upstream of OT cells become fluorescent, and an encoded indicator expressed in glutamatergic (VGLUT2-expressing) neurons. Sagittal periventricular acute slices in such brains may reveal parts of the rhythmogenic network driving OT neurons. In sum, the possibilities offered are many (Candlish et al., 2015; Sternson et al., 2016), provided that the *in vivo* electrical properties of OT neurons in nursing lactating mice are known, which will require repetition of a substantial amount of work in this animal species. The same approaches could allow refined analyses in the organotypic culture model, as well as *in vivo* studies including direct monitoring of cell activity using deep-brain imaging from the third ventricle in conscious animals (Sternson et al., 2016). All this applying to VP neurons in relation with their osmoregulatory networks, their position in the brain and relation to the blood-brain-barrier forming glia.

In conclusion, whatever the exact architecture of the systems driving the magnocellular neurons, and to what extent generalization will be possible, these hypothalamic cells keep offering renewed opportunities to advance our knowledge of the mechanisms of neuroendocrine regulations and peptidergic neurotransmission.

## Author contributions

JI performed experiments. PC initiated the project. JI, SO, and PC analyzed data, discussed the results and contributed to the writing of the manuscript.

## Funding

This research was supported by INSERM, La Fondation pour la Recherche Médicale (Equipe FRM grant to SO) and the Agence Nationale pour la Recherche (ANR, France; grant ANR 11-BSV1-021-lipobrain-03 to PC).

### Conflict of interest statement

The authors declare that the research was conducted in the absence of any commercial or financial relationships that could be construed as a potential conflict of interest.
